# Effect of Remote Ischaemic Conditioning on the Inflammatory Cytokine Cascade of COVID-19 (RIC in COVID-19): a Randomized Controlled Trial

**DOI:** 10.1007/s10557-022-07411-2

**Published:** 2022-11-29

**Authors:** Kishal Lukhna, Helison R. P. do Carmo, Alejandro Rossell Castillo, Sean M. Davidson, Hayli Geffen, Sara Giesz, Pelin Golforoush, Ticiane Gonçalez Bovi, Diana Gorag, Alan Salama, Aqeela Imamdin, Siavash Kalkhoran, Sandrine Lecour, Mauricio W. Perroud Jr., Mpiko Ntsekhe, Andrei C. Sposito, Derek M. Yellon

**Affiliations:** 1 Division of Cardiology, Faculty of Health Sciences, Groote Schuur Hospital, University of Cape Town, Cape Town, South Africa; 2 Atherosclerosis and Vascular Biology Laboratory, State University of Campinas, Campinas, Brazil; 3 The Hatter Cardiovascular Institute, University College London, 67 Chenies Mews, London, WC1E 6HX UK; 4 Cape Heart Institute, University of Cape Town, Cape Town, South Africa; 5 The Royal Free Hospital, University College London, Pond St, London, NW3 2QG UK

**Keywords:** COVID-19, Remote ischaemic conditioning, Randomized controlled trial, Cytokine storm, Immunosuppression, Inflammatory cascade

## Abstract

**Purpose:**

Patients hospitalized with COVID-19 may develop a hyperinflammatory, dysregulated cytokine “storm” that rapidly progresses to acute respiratory distress syndrome, multiple organ dysfunction, and even death. Remote ischaemic conditioning (RIC) has elicited anti-inflammatory and cytoprotective benefits by reducing cytokines following sepsis in animal studies. Therefore, we investigated whether RIC would mitigate the inflammatory cytokine cascade induced by COVID-19.

**Methods:**

We conducted a prospective, multicentre, randomized, sham-controlled, single-blind trial in Brazil and South Africa. Non-critically ill adult patients with COVID-19 pneumonia were randomly allocated (1:1) to receive either RIC (intermittent ischaemia/reperfusion applied through four 5-min cycles of inflation (20 mmHg above systolic blood pressure) and deflation of an automated blood-pressure cuff) or sham for approximately 15 days. Serum was collected following RIC/sham administration and analyzed for inflammatory cytokines using flow cytometry. The endpoint was the change in serum cytokine concentrations. Participants were followed for 30 days.

**Results:**

Eighty randomized participants (40 RIC and 40 sham) completed the trial. Baseline characteristics according to trial intervention were overall balanced. Despite downward trajectories of all cytokines across hospitalization, we observed no substantial changes in cytokine concentrations after successive days of RIC. Time to clinical improvement was similar in both groups (HR 1.66; 95% CI, 0.938–2.948, *p* 0.08). Overall RIC did not demonstrate a significant impact on the composite outcome of all-cause death or clinical deterioration (HR 1.19; 95% CI, 0.616–2.295, *p* = 0.61).

**Conclusion:**

RIC did not reduce the hypercytokinaemia induced by COVID-19 or prevent clinical deterioration to critical care.

**Trial Registration:**

ClinicalTrials.gov Identifier: NCT04699227.

**Supplementary Information:**

The online version contains supplementary material available at 10.1007/s10557-022-07411-2.

## Introduction

The coronavirus-19 disease (COVID-19) emerged in late 2019 and has since placed an enormous strain on healthcare resources, particularly in low- to middle-income countries (LMIC). There are currently well over 500 million cases documented worldwide, with approximately 6 million deaths reported globally at the time of writing [1]. About 15% of infected adults are hospitalized with severe COVID-19 pneumonia, and a subset rapidly progresses to acute respiratory distress syndrome (ARDS), multiple organ dysfunction syndrome (MODS), and even death [2, 3]. Early studies have correlated the presence of a cytokine “storm” in patients with COVID-19 and severe outcomes [4]. Interleukin (IL)-6 and tumor necrosis factor (TNF)-α have been considered by many as major cytokine culprits in the pathogenesis of this COVID-19-induced hyper-inflammation [5, 6]. Cytokine “storm” syndrome is a complex release of multiple cytokines in response to pathogenic material, such as SARS-CoV-2 [2]. Considering the high mortality and elevated pro-inflammatory cascade in those that deteriorate, suppressing this cytokine “storm” phenomenon may dampen immune hyperactivity and serve as a complementary therapeutic strategy as we advance our strategies against COVID-19 [7–9]. Previous trials have demonstrated a close link between COVID-19 disease severity and elevated IL-6 levels; however, most IL-6 neutralizing agents, such as tocilizumab, have shown modest benefit in patients hospitalized with COVID-19 [10, 11]. Dexamethasone, which has broad immunosuppressive roles via diverse mechanisms of action, has been shown to reduce lung injury induced by a cytokine storm [12]. However, not all cytokines are pro-inflammatory. Extensive non-tailored immunosuppression may be considered a double-edged sword especially if the delicate cytoprotective balance is compromised. For example, interferon (IFN)-γ is an immunomodulatory agent stimulated during infection and essential for downregulating viral replication. Described as an integral part of the body’s innate antiviral defence system, over-suppression may be harmful [13, 14]. Identifying or repurposing therapies which target the COVID-19-induced cytokine storm while preserving the cytoprotective cascade is therefore essential in halting or reducing the severity of damage in patients with COVID-19 [2].

Several animal models have suggested that the novel, noninvasive, and highly feasible intervention known as remote ischaemic conditioning (RIC) can suppress inflammatory cytokines and elicit cytoprotective and anti-inflammatory benefits in sepsis [15–18]. Early repetitive or chronic RIC administration has shown benefits in reducing levels of inflammatory cytokines (such as TNF-α, IL-1β, and IL-6) following lipopolysaccharide-induced sepsis and has been associated with a reduction in mortality in mice [17]. In recent years, RIC has grown from being an innovative strategy for cardiovascular protection [19–21]. This emerging field of RIC has been thought to protect organs (e.g., brain, heart, lungs, and kidneys) against the harmful effect of ischemic/reperfusion injury through activation of cell survival pathways and modulation of inflammatory responses that improve mitochondrial function [22–25]. RIC is a safe treatment modality whereby a blood-pressure cuff is applied to the upper arm for repeated cycles of supra-systolic inflation and deflation (typically four 5-min cycles). This process of short cycles of nonlethal ischaemia of the upper limb, followed by reperfusion activates neurohormonal pro-survival mechanisms in the body to protect vital organs at remotely injured sites [16, 18, 26, 27]. Given the pro-inflammatory cytokine cascade elicited by COVID-19, we hypothesized that the therapeutic efficacy of RIC on cytokines induced in sepsis models might be translated to the hypercytokinaemia array seen in hospitalized patients with COVID-19.

Following the RECOVERY trial published in the latter half of 2020, dexamethasone, after demonstrating a significant mortality reduction in hypoxic hospitalized patients with COVID-19, has been rolled out in almost all centres worldwide [28]. It is possible that the immunomodulatory and cytoprotective effects stimulated by RIC, as additional therapy in our anti-COVID-19 artillery, could provide further benefit. However, the ability of RIC to modulate levels of inflammatory cytokines in hospitalized patients with COVID-19 remains to be established [18]. Here, we report the results of a preliminary proof-of-concept study designed to address this knowledge gap on the potential impact of RIC in patients with COVID-19.

## Methods

### Trial Design

We conducted an investigator-initiated, multicentre, international, single-blind, phase 3, parallel, randomized, sham-controlled trial to evaluate the effects of RIC on inflammatory cytokines in adults (aged ≥ 18 years) admitted to hospital with COVID-19 pneumonia. The trial was implemented in two low- to middle-income countries, Hospital Estadual de Sumaré in Campinas, Brazil, and Groote Schuur Hospital in Cape Town, South Africa (SA). Ethical approvals were obtained from regional health review boards at each participating hospital (State University of Campinas, Brazil: CAAE 33,709,320.4.0000.5404; and Human Research Ethics Committee, University of Cape Town, South Africa: HREC 407/2020) and were conducted in accordance with the principles of good clinical practice and accordance with the Declaration of Helsinki and its later amendments. “RIC in COVID-19” trial protocol was registered at ClinicalTrials.gov (identifier: NCT04699227) before the first randomization, and the study has been conducted and reported according to the CONSORT statement. All participants provided written informed consent before randomization. Details of the trial rationale and design have been previously published [29], and a copy of the protocol is available.

### Study Population

“RIC in COVID-19” included men and nonpregnant female patients with confirmed RT-PCR positive for SARS-CoV-2 at both recruiting centres. In addition, patients were included if they had a confirmed diagnosis of COVID-19 pneumonia on chest imaging and did not require critical care support, i.e., mechanical ventilation, vasopressors, or renal replacement therapy. Noninvasive respiratory support was defined as those requiring supplemental oxygen when delivered by nasal cannula, face mask, or high-flow nasal cannula respiratory support. Escalation of respiratory support was defined as the transition from noninvasive respiratory support to ventilation delivered by endotracheal or tracheostomy tube. Key exclusion criteria included contraindications to the usage of a brachial blood pressure cuff on either arm; intercurrent disease with a life expectancy of less than 24 h; recovery post-cardiac arrest; pregnant or breastfeeding women; bleeding disorders or platelet count below 50 × 10^9^/L; severe renal impairment (estimated glomerular filtration rate < 30 mL/min per 1.73 m^2^) or receipt of haemodialysis or peritoneal dialysis; chronic liver disease and/or ALT and AST ≥ 5 times the standard upper reference limit; significant immunodeficiency states: HIV/AIDS not on antiretroviral agents, solid organ transplants, and bone marrow transplants; chronic use of immunomodulating therapy such as TNF-α or chronic corticosteroids with prednisone-equivalent dose ≥ 20 mg/day; active underlying malignancy; symptomatic chronic obstructive pulmonary disease; baseline stage C chronic heart failure; and enrolment into any other investigational treatment study for COVID-19 in the 30 days before screening.

### Study Enrolment and Randomization

Eligible patients were enrolled within 24 h of hospital admission for acute COVID-19 pneumonia. All patients provided written informed consent. Participants were randomly assigned in a 1:1 ratio to receive either remote ischaemic conditioning or sham intervention. All participants received standard medical therapy according to national or local guidelines. Randomization was stratified by country, using a random permuted block size randomization sequence prepared by an independent statistician, and performed via a secure web-based clinical trial support system, i.e., REDCap [30], that was accessible 24 h a day. Randomization was performed by a designated study team member who was unblinded to the treatment allocation. Study participants, treating physicians, and study team members collecting data and assessing outcomes were blinded to treatment allocations.

### Trial Intervention

Automated preprogrammed pneumatic sphygmomanometer devices, sponsored by the University College London (UCL) and manufactured by Seagull Aps in Denmark, were used to deliver either the RIC or sham protocol throughout the trial. The RIC protocol comprised of applying a RIC device at enrolment on the upper arm to automatically deliver 4 cycles of 5-min sustained high-pressure cuff inflation (20 mmHg above each participant’s systolic blood pressure) alternating with 5-min sustained cuff deflation (0 mmHg), such that the total RIC protocol took 40 min. The sham protocol comprised the application of a visually identical pneumatic cuff on the upper arm, which automatically delivered sustained low-pressure cuff inflations (20 mmHg) and deflations of a similar frequency and duration as the RIC device. Participants received either RIC or sham on day 0 and were repeated daily for 15 days, or until clinical deterioration or discharge. An unblinded study team investigator applied the RIC and sham devices. Trained study investigators, independent of the treating physicians, assessed device-related adverse events and each participant’s clinical conditioning daily using the World Health Organization (WHO) ten-point Clinical Progression Scale (CPS) [31]. Safety assessments and clinical data were recorded on electronic case report forms that were validated by the trial’s quality control officer.

### Cytokine Sampling

Serum samples were prospectively collected from participants at baseline (before trial intervention and within 72 h of hospitalization) and every alternate day following the RIC/sham protocol, where possible, for the analysis of inflammatory cytokines. Samples were stored at − 80 °C and later thawed for batch cytokine analysis. We conducted a multiplex screen for 13 cytokines (IL-1β, IL-6, TNF-α, IP-10, IFN-λ1, IL-8, IL-12, IFN-α2, IFN-λ2/3, GM-CSF, IFN-β, IL-10, and IFN-γ) in a total of 80 COVID-19 participants, using the commercially available LEGENDplex™ Multi-Analyte Flow Assay kit according to the manufacturer’s protocol. Samples from days 0, 2, and 4 were selected as the most common days of sampling across the cohort and were analyzed in duplicate using flow cytometry (BD LSRII Fortessa; BD Biosciences, Franklin Lakes, NJ, USA) to detect cytokine levels. None of the study interventions or procedures delayed or affected the patient’s clinical management of COVID-19 pneumonia at each site.

### Study Endpoints

The primary endpoint of this trial was the median change of serum cytokines from admission (day 0) to the fourth day after inclusion. The prespecified secondary endpoints, analyzed in the intention-to-treat population, included (1) time to clinical deterioration (defined as time from randomization to mortality or a two-point reduction of the WHO Clinical Progression Scale, whichever came first) [31], (2) serum IL-6 ≥ 80 pg/mL as a biomarker for severe clinical outcomes in COVID-19 infection, and (3) cytokine score measured by longitudinal mixed-effects modelling. Safety endpoints included device-related adverse events, serious adverse events, and premature discontinuation of the trial intervention.

### Study Oversight

This trial was an academic research collaboration between the executive trial steering committee and investigators from UCL, UCT, and Unicamp. The academic research organization at each site coordinated data management. Statistical analyses were performed by the trial statistician using an independent copy of the complete raw dataset. The first version of the manuscript was drafted by the academic authors who take responsibility for the completeness and accuracy of the data and who made the decision to submit the manuscript for publication.

### Statistical Methods

Since there is no available data on the effect of RIC on the inflammatory cascade in patients with COVID-19, the sample size was therefore established empirically on 80 participants with a conservative expectation of a small, standardized effect size. The primary endpoint was compared between the RIC and sham groups across all 80 participants and between participants that had or had not deteriorated. The cytokine concentrations were analyzed separately in a longitudinal framework to determine the effect of the randomized treatment at baseline for each cytokine over time. The profile of cytokine concentrations over time exhibited both within-participant variabilities, resulting from repeated cytokine measurements over time for a single participant, and between-participant variabilities due to biological differences between participants included in the study. Therefore, to account for both within-participant and between-participant variabilities, linear mixed-effects models with discrete-time were employed to compare the effect of RIC versus sham on the cytokine concentrations over time. To address the primary aim of the study, the primary covariate, considered to be associated with cytokine concentrations, was the randomized baseline treatment of RIC or sham included as an interaction effect with discrete-time. To resolve between-participant variabilities for each cytokine response, random effects were imposed on the final model’s intercept and slope for discrete-time. The significance of the fixed effects in the model was assessed using conditional *t*-tests. To determine the random effects, all models were initially fit using maximum likelihood estimation, and model fit was evaluated using the Bayesian Information Criterion (BIC). The final model parameters were estimated using restricted maximum likelihood to obtain unbiased estimates of the variance components of the final model chosen by its BIC. The secondary time-to-event outcome was compared between the RIC and sham groups using Cox regression modelling stratified by the two components of the study on an intention-to-treat basis and presented with Kaplan–Meier curves to assess the total number of outcomes experienced by both groups up to 30 days post-hospital discharge. Further subgroup analysis of the primary outcome of death or deterioration was performed using a multivariate Cox proportional hazard model with a treatment-risk factor interaction included individually for each subgroup. The statistical analysis was performed in R, version 4.1.0.

### Data Availability

Data will be disclosed on request and approval of the proposed use by the trial steering committee. In addition, de-identified individual participant data will be made available, as well as data dictionaries and the study protocol. Data will be available for 5 years after the main study publication.

## Results

**Fig. 1:**
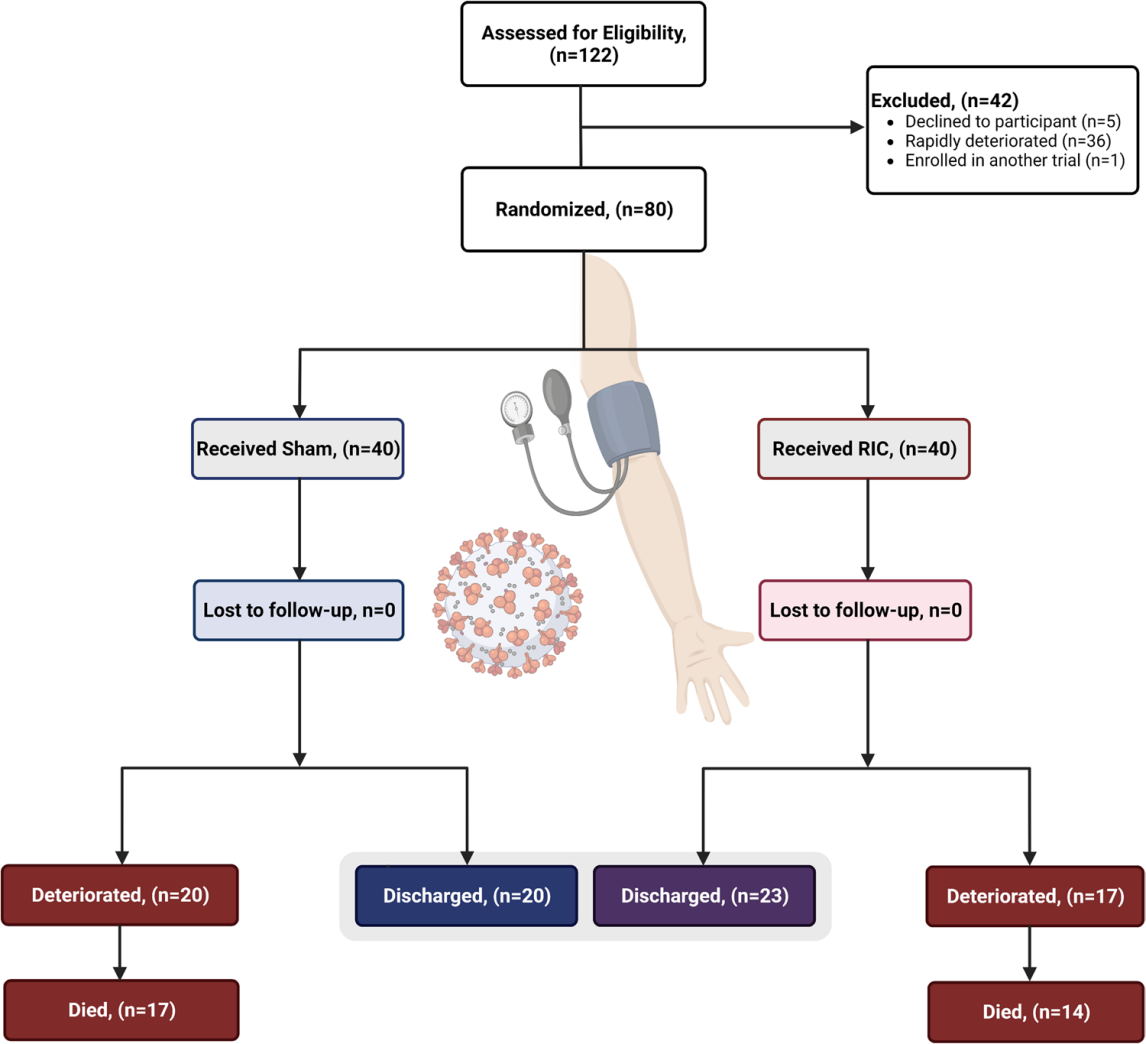
CONSORT flow diagram

**Fig. 2:**
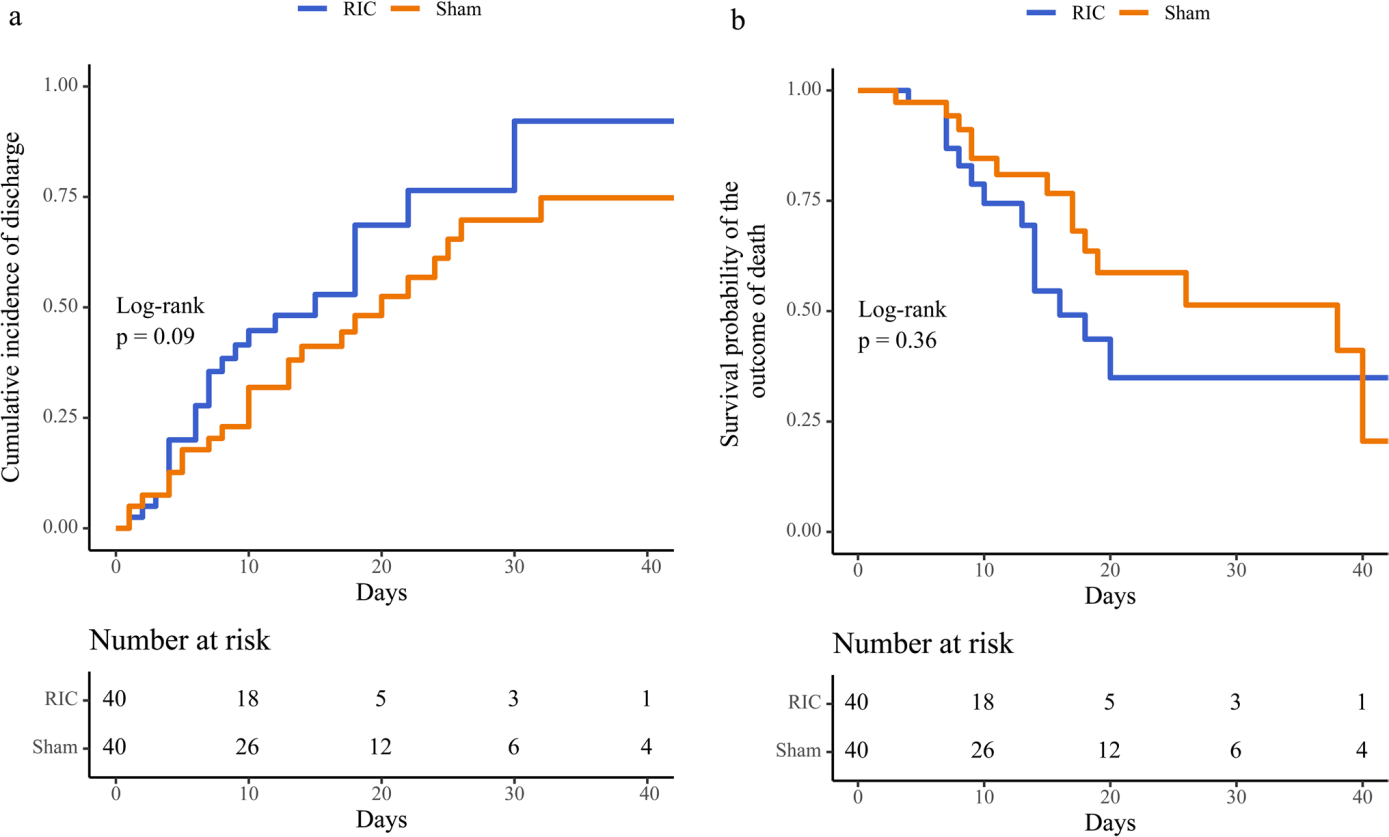
Survival analysis. Kaplan–Meier estimates of the cumulative incidence of discharge (**a**) and survival probability of the outcome of death (**b**)

Between January 15^th^, 2021, and August 31^st^, 2021, a total of 80 participants at 2 sites on either side of the Atlantic Ocean (40 in Brazil and 40 in South Africa) were randomly allocated. No participants withdrew from the study, and all participants were followed up for 30 days post-hospital discharge or death until a common study end date of October 31^st^, 2021. Forty participants were assigned and received RIC in the RIC treatment group, and 40 participants were assigned and received the sham intervention in the control treatment group. All 80 participants were included in the intention-to-treat analysis (Fig. 1). The RIC and sham interventions were completed according to the study protocol in all participants across both groups, and the results were included in the per-protocol analysis. No device-related adverse events from the RIC group were observed. Prior to randomization, 64 (80%) of 80 participants received systemic corticosteroids, including 34 (85%) of 40 participants who received RIC and 30 (75%) of 40 participants who received sham. Baseline characteristics according to the treatment groups were balanced and are summarized in Tables 1 and 2. The median age of the participants was 56 (IQR 50–67) years. A total of 48.8% of the participants were female, with a majority (68.8%) presenting with moderate COVID-19 disease (as defined by the WHO CPS severity scale) [31]. Obesity (63.7%) and hypertension (52.5%) were the most observed risk factors, with 32.5% having type 2 diabetes. Only 7.5% of the study population were HIV positive on antiretroviral therapy. The most common symptoms at presentation included cough (77.5%) and shortness of breath (87.5%), with the estimated average onset of symptoms beginning 9 (IQR 6–11) days before admission. All participants were admitted for hypoxemia requiring noninvasive respiratory support with a mean oxygen saturation of 93% (89–96%). Transition to invasive respiratory support occurred in 40% (32/80) of the trial population, with no significant differences between both treatment groups (*p* = 0.65). At discharge, 49 (61.25%) of 80 participants demonstrated clinical improvement, a median of 5 days shorter in the RIC group (Fig. 2a) (hazard ratio, 1.66; 95% CI, 0.938 to 2.948; *p* = 0.08). The composite outcome of clinical deterioration or death occurred in 37 (46.3%) participants. Overall, there were no significant differences between the study groups and the probability of death (Fig. 2b) (hazard ratio, 1.35; 95% CI, 0.650 to 2.776; *p* = 0.41). At follow-up, compared to sham, RIC had no significant impact on the composite outcome of all-cause death or clinical deterioration (hazard ratio, 1.19; 95% CI, 0.616 to 2.295; *p* = 0.61).

**Table 1:** Baseline characteristics of participants stratified according to treatment group

Treatment arm				
	Overall (*n* = 80)	Sham (*n* = 40)	RIC (*n* = 40)	*p* value
Age (years) (± IQR)	56.0 (50.0–67.0)	65.0 (54.0–68.0)	55.0 (48.5–63.3)	0.05
Female (%)	39 (48.8)	19 (47.5)	20 (50.0)	0.82
Oxygen saturation (%)	93.0 (89.0–96.0)	93.0 (89.0–96.0)	94.0(89.0–97.0)	0.60
Days from symptoms onset to admission (± IQR)	9.0 (6.0–11.0)	9.0 (6.0–11.0)	7.0 (5.0–11.3)	0.31
Baseline WHO CPS score (%)				
Moderate (4–5)	55 (68.8)	27 (67.5)	28 (70.0)	
Severe (6)	25 (31.3)	13 (32.5)	12 (30.0)	0.81
Baseline steroid initiation (%)	64 (80.0)	30 (75.0)	34 (85.0)	0.26
CRP (*n* = 67)	113.0 (66.3–168.0)	113.0 (56.8–176.6)	110.9 (69.3–161.0)	0.93
CRP > 80 mg/mL (*n* = 41)	41 (61.2)	20 (57.1)	21 (65.6)	0.48
IL-6 > 80 pg/mL (%)	25 (31.2)	16 (40.0)	9 (22.5)	0.09
Median time to discharge (days) (95% CI)	18 (12–24)	20 (13–32)	15 (8–30)	0.09
Median time to death (days) (95% CI)	20 (17–48)	38 (18–NA)	16 (14–NA)	0.36
Median time to deterioration (days) (95% CI)	13 (12–NA)	13 (10–NA)	12 (8–NA)	0.61
Intubation (%)	32 (40.0)	17 (42.5)	15 (37.5)	0.65
Symptoms at presentation (%)				
Dyspnoea	70 (87.5)	37 (92.5)	33 (82.5)	0.18
Cough	62 (77.5)	33 (82.5)	29 (72.5)	0.28
Fever	60 (75.0)	31 (77.5)	29 (72.5)	0.61
Fatigue	49 (61.3)	23 (57.5)	26 (65.0)	0.49
Myalgia	25 (31.2)	14 (35.0)	11 (27.5)	0.47
Diarrhoea	8 (10.0)	4 (10.0)	4 (10.0)	0.99
Comorbidities (%)				
Hypertension	42 (52.5)	23 (57.5)	19 (47.5)	0.37
Type 2 diabetes	26 (32.5)	12 (30.0)	14 (35.0)	0.63
Dyslipidaemia	28 (35.0)	17 (42.5)	11 (27.5)	0.16
Obesity	51 (63.7)	26 (65.0)	25 (62.5)	0.82
Smoker	13 (16.3)	9 (22.5)	4 (10)	0.13
HIV	6 (7.5)	2 (5.0)	4 (10.0)	0.68
Baseline medication (%)				
Statins	15 (18.8)	11 (27.5)	4 (10.0)	0.08
Angiotensin receptor blockers	18 (22.5)	12 (30.0)	6 (15.0)	0.11
ACE-inhibitors	13 (16.2)	5 (12.5)	8 (20.0)	0.36
Beta-blockers	7 (8.8)	2 (5.0)	5 (12.5)	0.43
Diuretics	13 (16.2)	8 (20.0)	5 (12.5)	0.36
Metformin	14 (17.5)	7 (17.5)	7 (17.5)	0.99
Insulin	8 (10.0)	4 (10.0)	4 (10.0)	0.99

*p* value corresponds to the Mann–Whitney *U* test comparing each treatment group

IQR, interquartile range; CL, confidence level; CRP, C-reactive protein; IL, interleukin; ACE, angiotensin-converting enzyme

**Table 2:** Baseline characteristics of participants stratified according to country

	Overall (*n* = 80)	Brazil (*n* = 40)	South Africa (*n* = 40)	*p* value
Age (years) (± IQR)	56.0 (50.0–67.3)	56.0 (50.0–67.3)	55.0 (49.5–67.0)	0.67
Female (%)	39 (48.8)	18 (45.0)	21 (52.5)	0.50
Oxygen saturation (%)	93.0 (89.0–96.0)	96.0 (93.8–97.0)	89.0(88.0–92.3)	< 0.0001
Days from symptoms onset to admission (± SD)	9.0 (6.0–11.0)	10.0 (8.5–12.0)	7.0 (5.0–9.0)	< 0.0001
Baseline WHO CPS score (%)				
Moderate (4–5)	55 (68.75)	24 (60.00)	31 (77.50)	
Severe (6)	25 (31.25)	16 (40.00)	9 (22.50)	0.09
Baseline steroid initiation (%)	64 (80.0)	24 (60.0)	40 (100.0)	< 0.0001
CRP (*n* = 67)	113.0 (66.3–168.0)	123.5 (74.5–219.0)	85.0 (61.5–147.5)	0.09
CRP > 80 mg/mL	41 (61.19)	23 (69.70)	18 (52.94)	0.16
IL-6 > 80 pg/mL (%)	25 (31.5)	12 (30.0)	13 (32.5)	0.81
Median time to discharge (days) (95% CI)	18 (12–24)	18 (10–26)	18 (10–30)	0.84
Median time to death (days) (95% CI)	20 (17–48)	19 (15–NA)	38 (16–NA)	0.30
Median time to deterioration (days) (95% CI)	13 (12–NA)	12 (8–NA)	14 (12–NA)	0.06
Intubation (%)	32 (40.0)	24 (60.0)	8 (20.0)	0.0003
Symptoms at presentation (%)				
Dyspnoea	70 (87.5)	35 (87.5)	35 (87.5)	0.99
Cough	62 (77.5)	34 (85.0)	28 (70.0)	0.11
Fever	60 (75.0)	29 (72.5)	31 (77.5)	0.61
Fatigue	49 (61.3)	26 (65.0)	23 (57.5)	0.49
Myalgia	25 (31.2)	12 (30.0)	13 (32.5)	0.81
Diarrhoea	8 (10.0)	4 (10.0)	4 (10.0)	0.99
Comorbidities (%)				
Hypertension	42 (52.5)	20 (50.0)	22(55.0)	0.65
Type 2 diabetes	26 (32.5)	11 (28.0)	15 (37.5)	0.34
Dyslipidaemia	28 (35.0)	8 (20.0)	20 (50.0)	0.01
Obesity	51 (63.7)	22 (55.0)	29 (72.5)	0.10
Smoker	13 (16.3)	8 (20.0)	5 (12.5)	0.36
HIV	6 (7.5)	0 (0)	6 (15.0)	0.03
Baseline medication (%)				
Statins	15 (18.8)	8 (20.0)	7 (17.5)	0.78
Angiotensin receptor blockers	18 (22.5)	15 (37.5)	3 (7.5)	0.003
ACE-inhibitors	13 (16.2)	4 (10.0)	9 (22.5)	0.23
Beta-blockers	7 (8.8)	6 (15.0)	1 (2.5)	0.11
Diuretics	13 (16.2)	5 (12.5)	8 (20.0)	0.36
Metformin	14 (17.5)	8 (20.0)	6 (15.0)	0.56
Insulin	8 (10.0)	4 (10.0)	4 (10.0)	0.99

*p* value corresponds to the Mann–Whitney *U* test comparing each treatment group

IQR, interquartile range; CL, confidence level; CRP, C-reactive protein; IL, interleukin; ACE, angiotensin-converting enzyme

### Cytokine Analysis

**Fig. 3:**
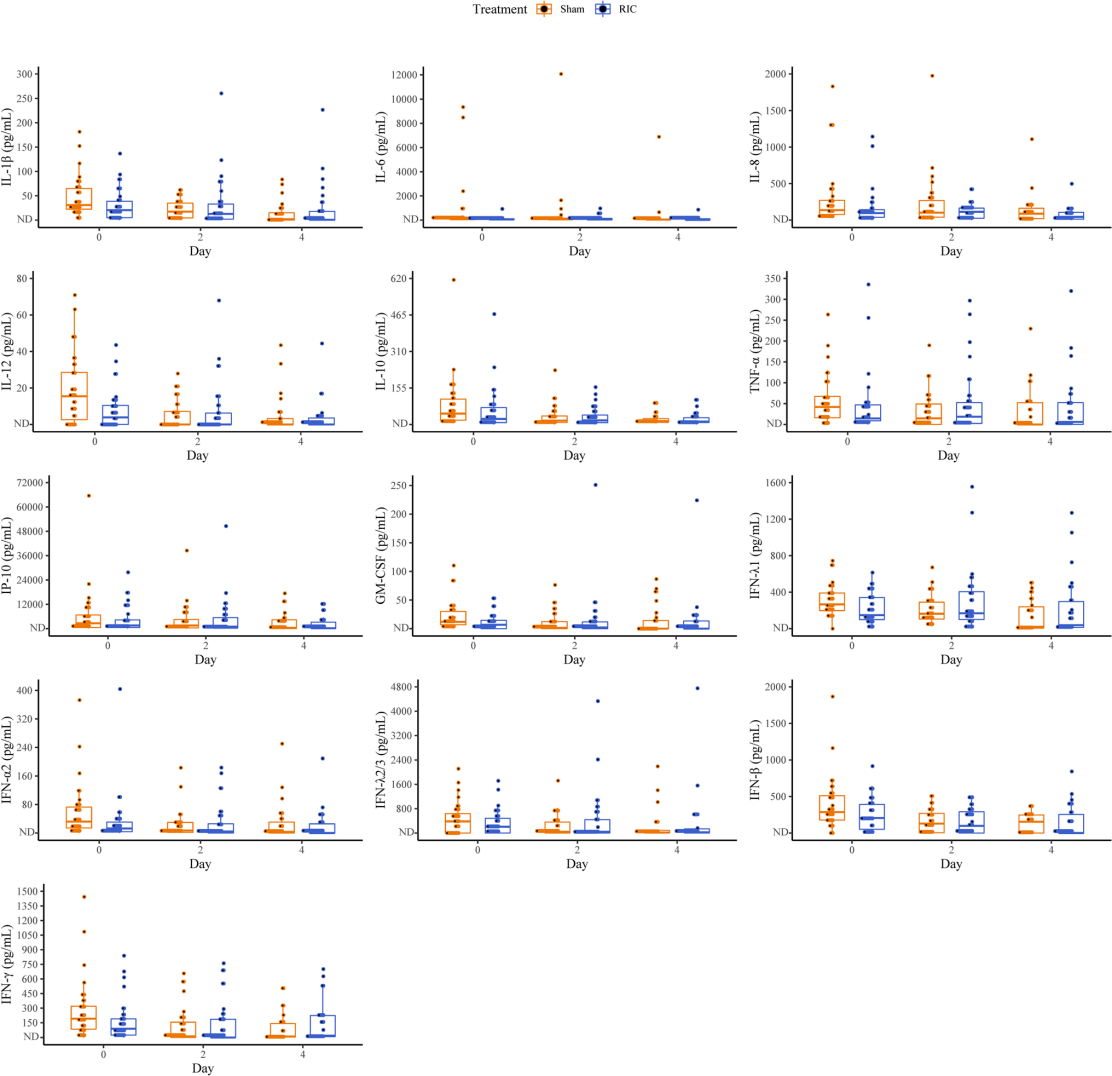
All cytokines. Individual cytokine concentrations over time stratified by each treatment group

We conducted a multiplex screen for 13 cytokines in all 80 participants. We monitored the effects of RIC and sham on the selected inflammatory cascade from baseline to the second and fourth days of hospitalization, presented in Table 3. Variances of each cytokine undergoing RIC and sham intervention were further assessed between those that had deteriorated requiring critical care support and those that did not and presented in Tables 4 and 5. By utilizing linear mixed-effects modelling with discrete-time to adjust for both within-participant and between-participant cytokine variabilities at baseline, we were able to account for and further compare the effects of RIC and sham on all 13 immune cytokine profiles. As shown, the proportional and absolute between-group differences in cytokine concentrations varied considerably at baseline and across hospitalization. Despite the downward trajectories of all cytokines across hospitalization, there were no significant differences between the median change in cytokine concentrations from baseline in those who received the RIC intervention compared to sham with respect to the primary endpoint on day 2 or day 4. IL-8 showed a greater reduction from baseline to day 4 in the RIC group; however, it demonstrated a significant difference in baseline concentrations between both groups. Contrastingly, TNF-α showed a relative increase from baseline to day 4 in both groups. At baseline, 43.62% (35/80) of the cohort presented with IL-6 values > 80 pg/mL, of which 54.3% deteriorated or died. Although not significant, IL-6 concentrations were observed to be higher across both arms in the subgroup of participants that deteriorated compared to those that did not, with lower IL-6 values observed on day 4 after receiving RIC in the group of participants that did not require critical support (Table 5). Finally, IL-10 and IL-12 showed similar variations across both groups, with baseline values higher in the sham group, subsequently affecting the median change in cytokine concentrations during analysis (Fig. 3).

**Table 3:** Change in cytokine concentration (pg/mL) from baseline, stratified according to treatment group, *n* = 80

Cytokine	Day	Range	*Number detectable (%)	Sham	RIC	*p* value
IL-1β	Baseline	ND–181.52	69 (87.34)	31.17 (23.01–65.08)	23.40 (18.48–47.49)	0.14
	2	ND–260.30	69 (88.46)	21.35 (8.34–38.24)	15.18 (3.57–37.25)	0.48
	4	ND–226.61	39 (60.94)	13.09 (1.69–23.98)	17.07 (4.35–47.61)	0.32
IL-6	Baseline	ND–9344.07	77 (97.47)	123.38 (34.19–242.90)	60.72 (28.55–109.87)	0.02
	2	ND–12068.31	75 (96.15)	67.75 (35.03–158.79)	75.19 (18.07–157.92)	0.62
	4	ND–6884.12	62 (96.88)	44.83 (17.74–84.05)	24.13 (9.15–120.29)	0.44
TNF-α	Baseline	ND–335.53	69 (87.34)	49.11 (19.14–68.90)	22.69 (11.19–48.42)	0.01
	2	ND–296.79	60 (76.92)	33.49 (10.58–59.31)	37.82 (5.46–60.69)	0.94
	4	ND–319.92	31 (48.44)	55.89 (38.20–102.83)	49.76 (20.85–75.95)	0.47
IP-10	Baseline	81.82–65,555.21	79 (100)	2659.83 (566.58–6769.89)	1557.56 (550.00–4338.57)	0.31
	2	20.87–50,575.64	78 (100)	1558.52 (380.63–4505.26)	1008.29 (250.60–5450.85)	0.64
	4	20.64–17,413.14	64 (100)	371.18 (69.25–4370.33)	227.31 (68.17–3225.64)	0.50
IFN-λ1	Baseline	ND–744.48	75 (94.94)	267.69 (204.64–391.74)	187.14 (113.03–346.06)	0.01
	2	ND–1554.84	77 (98.72)	163.55 (104.90–290.31)	170.28 (106.76–406.89)	0.66
	4	ND–1269.26	49 (76.56)	180.40 (17.01–330.02)	142.54 (23.02–414.32)	0.68
IL-8	Baseline	ND–1829.22	78 (98.73)	136.39 (76.58–270.79)	96.78 (41.81, 142.85)	0.03
	2	ND–1974.92	77 (98.72)	102.35 (39.72–268.17)	115.00 (31.36, 164.50)	0.46
	4	ND–1108.30	61 (95.31)	94.24 (31.56–179.28)	46.79 (15.96, 109.89)	0.13
IL-12	Baseline	ND–70.90	52 (65.82)	18.89 (13.66–33.35)	9.95 (6.94–14.44)	0.002
	2	ND–67.92	34 (43.59)	8.86 (6.22–17.38)	10.47 (3.74–20.55)	0.97
	4	ND–44.35	21 (32.81)	7.02 (4.19–16.31)	5.78 (3.59–11.17)	0.61
IFN-α2	Baseline	ND–403.93	66 (83.54)	34.52 (16.81–76.30)	19.11 (8.06–40.27)	0.04
	2	ND–183.58	52 (66.67)	13.79 (4.15–30.49)	19.50 (8.88–56.06)	0.15
	4	ND–250.34	36 (56.25)	15.78 (4.05–53.81)	26.76 (8.01–52.78)	0.54
IFN-λ2/3	Baseline	ND–2112.20	53 (67.09)	560.56 (428.62–837.44)	273.56 (204.45–648.01)	0.01
	2	ND–4330.71	50 (64.10)	241.64 (52.35–523.39)	137.77 (43.04–727.26)	0.60
	4	ND–4752.88	36 (56.25)	80.07 (40.37–437.27)	96.50 (36.82–598.68)	0.74
GM-CSF	Baseline	ND–110.28	53 (67.09)	18.75 (9.89–33.90)	13.25 (8.04–23.70)	0.26
	2	ND–251.04	41 (52.56)	11.97 (3.84–29.63)	10.98 (6.75–20.09)	0.99
	4	ND–224.13	21 (32.81)	38.21 (18.02–66.73)	20.64 (15.20–25.67)	0.28
IFN-β	Baseline	ND–1866.95	67 (84.81)	292.16 (192.94–515.56)	226.07 (194.89–438.60)	0.41
	2	ND–513.38	56 (71.79)	180.10 (119.34–313.62)	230.64 (89.36–342.97)	0.55
	4	ND–843.36	33 (51.56)	238.54 (188.65–262.47)	315.92 (142.02–411.28)	0.27
IL-10	Baseline	ND–613.82	78 (98.73)	46.18 (18.17–107.32)	25.33 (9.72–74.96)	0.08
	2	ND–230.25	75 (96.15)	16.92 (9.84–37.96)	17.52 (8.10–41.82)	0.90
	4	ND–106.55	62 (96.88)	13.88 (8.85–24.67)	14.92 (8.93–30.31)	0.71
IFN-γ	Baseline	ND–1443.09	64 (81.01)	223.25 (124.58–349.93)	127.75 (70.32–227.06)	0.04
	2	ND–761.07	36 (46.15)	163.60 (82.12–247.17)	197.96 (174.61–540.77)	0.21
	4	ND–701.25	57 (89.06)	13.17 (7.56–156.29)	21.48 (9.21–226.08)	0.27

*p* value corresponds to the Mann–Whitney *U* test comparing the average change in cytokine levels across the treatment groups

ND, non-detectable

^*^Number detectable represents cytokine concentrations detected above the lower limit of detection (LoD) of the LEGENDplex™ Multi-Analyte Flow Assay kit

## Discussion

In this first study of its kind designed to assess the impact of RIC on inflammatory cytokines in participants admitted to hospital with COVID-19, we were unable to detect a significant reduction by RIC in levels of pro-inflammatory cytokines (including IL-1β, IL-6, and TNF-α). Furthermore, RIC did not prevent clinical deterioration or reduce mortality at 30 days.

The cytokine storm induced by COVID-19 served as a novel target for RIC, which has been found to influence multiple pro-survival pathways in a number of clinical settings [16, 18, 26, 27]. In order to test the potential impact of RIC in the setting of COVID-19, we selected a panel of pro-inflammatory cytokines that included IL-6, TNF-α, IL-1β, and IFN-γ. Although RIC has been shown to lower levels of IL-6, TNF-α, and IL-1β [15, 17] and improve survival in animal models of sepsis, whether this concept inspired by animal studies has an impact on inflammatory cytokines stimulated by COVID-19 has not been previously explored. Almost half of our cohort presented with IL-6 concentrations > 80 pg/mL, a prognosticating value associated with respiratory failure in patients with COVID-19 [11]. Baseline levels of other important cytokines of interest were also variably elevated. In the early stages of the COVID-19 pandemic, a study by Huang et al. revealed that elevated circulating levels of IL-6 were associated with clinical deterioration and the need for critical care support, suggesting that IL-6 could potentially serve as a target for intervention [4, 32]. Although the prognostic value of TNF-α and IL-1β as therapeutic targets in COVID-19 is not known [33–35], they are important makers of disease severity in other infectious and inflammatory conditions [36–38]. Despite marked cytokine levels at presentation, the overall clinical outcomes of participants in both our interventional and control groups were similar, and we were not able to detect any significant interaction with RIC.

The drivers of the severe hypercytokinaemia noted during fatal COVID-19 infections remain ill-understood and are likely influenced by both host- and virus-related factors [33, 39]. This is important because, in our study, there were significant differences in baseline characteristics, comorbidities, and measures of disease severity between recruiting centres. In particular, participants from South Africa had a higher frequency of the metabolic syndrome (dyslipidaemia, type 2 diabetes, and obesity) and HIV infection compared to participants in Brazil. Significant differences were also noted in baseline oxygenation and symptom onset between participants at both sites. Those from SA presented earlier with lower oxygen saturation levels compared to participants from Brazil. Although these differences may have influenced both the cytokine profile and the cytoprotective signalling induced by RIC, no significant interactions were found between baseline characteristics or clinical variables and the impact of RIC.

Among patients with COVID-19, several phases of the disease are described, each associated with different cytokine release profiles [40–42]. This is important because the marked variation in measured cytokines both at baseline and throughout the study may be accounted for by the fact that the phase at presentation was not known. We attempted to account for within-participant, between-participants, and between-site differences in cytokine profiles by using linear mixed-effects modelling to analyze the impact of RIC versus sham over time. However, whether this was adequate to mitigate against the impact of this phase effect is not clear. Therefore, enrolling participants in different phases of their disease may in part explain our findings. While the aforementioned limitations may not have fully unmasked a positive signal from RIC, our study has been very helpful in identifying and validating potential pitfalls for success as described by Bell et al. [19] and highlights the need to further investigate the easily accessible and cost-effective benefits of RIC in patients with COVID-19. Given the involvement of ongoing inflammation with possible cytokine elevation in patients with long COVID-19 and the paucity of specific treatments for this syndrome, it may be interesting to study the potential benefit of RIC in this population [ 43. ].

## Limitations

This study has several limitations that need to be considered when evaluating the findings. Firstly, as a pilot study, the sample size could not be calculated a priori, limiting the statistical power for some outcomes. In addition, as we did not set any scale to determine the severity of non-critically ill patients enrolled at hospital admission, many participants displayed a wide variation in symptom onset. Furthermore, our study was designed and conducted during an era of the pandemic when the optimal treatment strategy for patients with COVID-19 was rapidly evolving. For this reason, corticosteroid initiation was not mandatory before enrolment and left to the treating physician’s discretion, and as a result, varied significantly between sites. These factors may have contributed to the extensive variation in cytokine concentrations and made it difficult to evaluate the effect of RIC. This later point is important as it clearly affected our secondary endpoint, which was to test RIC as an adjunct to standard of care. In addition, a critical limitation was encountered during cytokine analysis where a substantial amount of cytokine concentrations across hospitalization were found undetectable as they fell below the standardized kit’s detection level and, as a result, could not be included in the analysis. Finally, the study was carried out in the early phase of the pandemic with mostly unvaccinated participants. Therefore, care should be taken when extrapolating these findings to contemporary patients vaccinated and exposed to different strains of SARS-CoV-2.

## Conclusion

Compared to sham, RIC did not reduce the in-hospital inflammatory cytokine cascade associated with moderate-and-severe COVID-19 and did not mitigate clinical deterioration. The findings from the “RIC in COVID-19” trial have highlighted the need for further research into the understanding of RIC and the dysregulated hyperinflammatory spectrum induced by SARS-CoV-2.

## Supplementary Information

Below is the link to the electronic supplementary material.

Supplementary file1 (DOCX 44 kb)

## Data Availability

Requests for data collected for the study can be made to the co-corresponding author and will be considered individually. Additional related documents are immediately available (e.g., study protocol and informed consent form) and can be requested from the co-corresponding author.
