# Supplementary material for: Effect of Remote Ischaemic Conditioning on the Inflammatory Cytokine Cascade of COVID-19 (RIC in COVID-19): a Randomized Controlled Trial
Source: Cardiovasc Drugs Ther. 2022 Nov 29;38(3):433–45. doi: 10.1007/s10557-022-07411-2 (PMC9707178; doi:10.1007/s10557-022-07411-2)
Supplement: Supplementary file 1 — Supplementary file1 (DOCX 44 kb) [file 10557_2022_7411_MOESM1_ESM.docx]

**Supplementary Information: Tables 4-6**

| Table 4. Change in cytokine concentration (pg/mL) from baseline, stratified according to treatment group in those that deteriorated or died, n=37 | | | | | | |
| --- | --- | --- | --- | --- | --- | --- |
|  | | | | |  |  |
| Cytokine | **Day** | **Range** | **Number detectable (%)** | **Sham** | **RIC** | **p-value** |
| IL-1β | Baseline | ND - 181.52 | 31 (83.78) | 39.01 (20.33 - 74.88) | 22.73 (20.06 - 42.21) | 0.33 |
|  | 2 | ND - 82.83 | 33 (89.19) | 17.17 (4.16 - 34.28) | 14.45 (3.16 - 26.63) | 0.58 |
|  | 4 | ND - 50.38 | 13 (44.83) | 6.11 (1.38 - 12.71) | 16.03 (15.06 - 22.05) | 0.09 |
|  | | | | | | |
| IL-6 | Baseline | 4.24 - 9344.07 | 37 (100) | 150.89 (61.84 - 323.49) | 66.58 (29.32 - 118.83) | 0.07 |
|  | 2 | ND - 1638.29 | 36 (97.30) | 50.58 (31.34 - 170.37) | 125.43 (73.44 - 227.66) | 0.24 |
|  | 4 | 4.74 - 303.34 | 29 (100) | 35.08 (16.44 - 62.08) | 60.46 (15.06 - 135.48) | 0.59 |
|  | | | | | | |
| TNF-α | Baseline | ND - 335.53 | 31 (83.78) | 47.34 (17.07 - 66.04) | 14.52 (10.26 - 37.94) | 0.05 |
|  | 2 | ND - 162.57 | 26 (70.27) | 21.20 (4.86 - 37.22) | 43.34 (13.22 - 49.16) | 0.34 |
|  | 4 | ND - 86.30 | 12 (41.38) | 35.94 (27.21 - 43.50) | 44.00 (22.57 - 59.05) | 0.81 |
|  | | | | | | |
| IP-10 | Baseline | 81.82 - 27781.03 | 37 (100) | 2453.51 (827.21 - 4587.39) | 1703.04 (691.30 - 3766.14) | 0.92 |
|  | 2 | 20.87 - 50575.64 | 37 (100) | 877.58 (257.38 - 1920.41) | 1432.84 (820.41 - 6440.76) | 0.10 |
|  | 4 | 32.38 - 12893.60 | 29 (100) | 123.02 (69.25 - 906.62) | 266.57 (92.25 - 1636.42) | 0.35 |
|  | | | | | | |
| IFN-λ1 | Baseline | ND - 709.71 | 36 (97.30) | 310.08 (190.44 - 452.80) | 311.88 (132.83 - 420.42) | 0.46 |
|  | 2 | ND - 671.47 | 36 (97.30) | 135.94 (101.54 - 293.80) | 246.27 (141.58 - 419.54) | 0.12 |
|  | 4 | ND - 726.99 | 24 (82.76) | 24.52 (15.92 - 226.28) | 132.69 (17.97 - 443.69) | 0.52 |
|  | | | | | | |
| IL-8 | Baseline | 28.61 - 1829.22 | 37 (100) | 195.91 (83.75 - 417.05) | 98.380 (42.02 - 143.43) | 0.10 |
|  | 2 | 2.59 - 1974.92 | 37 (100) | 92.23 (41.57 - 324.14) | 160.18 (112.09 - 173.96) | 0.19 |
|  | 4 | 8.83 - 181.57 | 29 (100) | 36.98 (25.65 - 92.91) | 53.00 (36.95 - 102.69) | 0.38 |
|  | | | | | | |
| IL-12 | Baseline | ND - 63.05 | 27 (72.97) | 19.67 (13.63 - 39.33) | 9.34 (7.02 - 14.90) | 0.02 |
|  | 2 | ND - 35.96 | 14 (37.84) | 6.89 (6.16 - 11.55) | 3.62 (3.38 - 10.03) | 0.52 |
|  | 4 | ND - 43.42 | 7 (24.14) | 6.54 (5.52 - 15.89) | 4.01 (3.83 - 4.98) | 0.40 |
|  | | | | | | |
| IFN-α2 | Baseline | ND - 403.93 | 32 (86.49) | 46.87 (15.30 - 80.15) | 14.03 (7.93 - 34.56) | 0.12 |
|  | 2 | ND - 167.87 | 26 (70.27) | 9.18 (2.99 - 29.62) | 11.79 (6.39 - 39.66) | 0.47 |
|  | 4 | ND - 96.63 | 16 (55.17) | 6.34 (3.24 - 10.82) | 27.84 (8.21 - 48.68) | 0.25 |
|  | | | | | | |
| IFN-λ2/3 | Baseline | ND - 1716.30 | 24 (64.86) | 556.24 (437.19 - 720.35) | 401.64 (224.96 - 778.50) | 0.47 |
|  | 2 | ND - 1020.98 | 24 (64.86) | 136.32 (51.47 - 363.66) | 424.21 (44.55 - 736.22) | 0.38 |
|  | 4 | ND - 1018.39 | 14 (48.28) | 49.40 (26.50 - 80.07) | 134.69 (96.50 - 161.10) | 0.15 |
|  | | | | | | |
| GM-CSF | Baseline | ND - 110.28 | 25 (67.57) | 18.99 (10.43 - 37.12) | 18.22 (8.90 - 36.97) | 0.72 |
|  | 2 | ND - 48.08 | 17 (45.95) | 12.22 (4.65 - 32.90) | 14.10 (6.31 - 17.05) | 0.96 |
|  | 4 | ND - 69.71 | 9 (31.03) | 17.59 (10.76 - 68.69) | 17.28 (12.26 - 21.71) | 0.73 |
|  | | | | | | |
| IFN-β | Baseline | ND - 1866.95 | 31 (83.78) | 322.39 (225.32 - 662.94) | 392.40 (214.73 - 457.71) | 0.86 |
|  | 2 | ND - 513.38 | 26 (70.27) | 224.72 (112.85 - 314.24) | 258.08 (97.74 - 433.90) | 0.44 |
|  | 4 | ND - 534.87 | 14 (48.28) | 218.45 (104.09 - 242.35) | 394.30 (257.91 - 428.13) | 0.07 |
|  | | | | | | |
| IL-10 | Baseline | 3.39 - 613.82 | 37 (100) | 64.10 (17.12 - 129.59) | 29.04 (6.22 - 83.34) | 0.33 |
|  | 2 | ND - 230.25 | 35 (94.59) | 16.92 (9.75 - 39.98) | 23.56 (11.30 - 65.49) | 0.46 |
|  | 4 | ND - 99.22 | 28 (96.55) | 14.79 (9.63 - 24.67) | 18.69 (12.20 - 28.38) | 0.31 |
|  | | | | | | |
| IFN-γ | Baseline | ND - 838.72 | 29 (78.38) | 196.29 (86.70 - 303.77) | 160.28 (87.63 - 253.57) | 0.71 |
|  | 2 | ND - 712.52 | 15 (40.54) | 157.64 (105.16 - 221.86) | 189.22 (179.13 - 262.42) | 0.34 |
|  | 4 | ND - 627.19 | 25 (86.21) | 9.37 (6.61 - 30.54) | 18.23 (10.25 - 173.69) | 0.05 |
|  | | | | | | |

*P-value* *corresponds to the Mann-Whitney U test comparing the average change in cytokine levels across the treatment groups.*

*ND=Non-Detectable*

**ND=Number detectable, represents cytokine concentrations detected above the lower limit of detection (LoD) of the LEGENDplex™ Multi-Analyte Flow Assay kit.*

| Table 5. Change in cytokine concentration (pg/mL) from baseline, stratified according to treatment group in those that did not deteriorate, n=43 | | | | | | |
| --- | --- | --- | --- | --- | --- | --- |
|  | | | | |  |  |
| Cytokine | **Day** | **Range** | **Number detectable (%)** | **Sham** | **RIC** | **p-value** |
| IL-1β | Baseline | ND – 152.31 | 38 (90.48) | 30.66 (23.78 - 54.52) | 24.75 (17.09 - 56.73) | 0.43 |
|  | 2 | ND – 260.30 | 36 (87.80) | 24.44 (12.40 - 47.78) | 15.18 (4.17- 67.46) | 0.66 |
|  | 4 | ND - 226.61 | 26 (74.29) | 14.33 (12.06 - 32.95) | 17.99 (2.95 - 66.49) | 0.84 |
|  | | | | | | |
| IL-6 | Baseline | ND - 8484.17 | 40 (95.24) | 87.67 (27.59 - 209.91) | 57.33 (25.22 - 88.16) | 0.24 |
|  | 2 | ND - 12068.31 | 39 (95.12) | 73.15 (47.43 - 153.90) | 29.29 (13.69 - 114.23) | 0.08 |
|  | 4 | ND - 6884.120 | 33 (94.29) | 56.37 (24.67 - 103.96) | 13.96 (6.60 - 64.38) | 0.19 |
|  | | | | | | |
| TNF-α | Baseline | ND - 263.52 | 38 (90.48) | 49.11 (21.68 - 106.05) | 23.12 (12.00 - 51.73) | 0.15 |
|  | 2 | ND - 296.79 | 34 (82.93) | 49.10 (33.49 - 74.48) | 35.89 (4.66 - 67.59) | 0.26 |
|  | 4 | ND - 319.92 | 19 (54.29) | 59.41 (53.07 - 104.46) | 51.31 (23.58 - 142.79) | 0.50 |
|  | | | | | | |
| IP-10 | Baseline | 102.38 - 65555.21 | 42 (100) | 4019.61 (505.54 - 10042.49) | 1094.01 (470.62 - 5990.61) | 0.29 |
|  | 2 | 41.104 - 38521.31 | 41 (100) | 3011.54 (1037.73 - 8561.03) | 710.76 (107.69 - 3732.17) | 0.04 |
|  | 4 | 20.64 - 17413.14 | 35 (100) | 3978.90 (201.82 - 5235.24) | 82.33 (45.15 - 3972.70) | 0.14 |
|  | | | | | | |
| IFN-λ1 | Baseline | ND - 744.48 | 39 (92.86) | 261.80 (217.78 - 360.84) | 137.96 (107.62 - 236.32) | 0.004 |
|  | 2 | 18.26 - 1554.84 | 41 (100) | 168.24 (126.01 - 290.31) | 158.03 (51.70 - 308.54) | 0.43 |
|  | 4 | ND - 1269.26 | 25 (71.43) | 215.95 (138.35 - 356.61) | 196.65 (32.77 - 326.21) | 0.94 |
|  | | | | | | |
| IL-8 | Baseline | ND - 497.28 | 41 (97.62) | 107.94 (54.72 - 201.57) | 96.25 (41.75 - 141.12) | 0.16 |
|  | 2 | ND - 714.99 | 40 (97.56) | 111.98 (38.68 - 207.24) | 54.05 (12.18 - 127.61) | 0.04 |
|  | 4 | ND - 1108.30 | 32 (91.43) | 159.19 (95.16 - 234.11) | 31.19 (9.15 - 108.88) | 0.02 |
|  | | | | | | |
| IL-12 | Baseline | ND - 70.90 | 25 (59.52) | 17.57 (13.71 - 28.01) | 10.81 (8.39 - 13.39) | 0.04 |
|  | 2 | ND - 67.92 | 20 (48.78) | 17.25 (7.00 - 19.63) | 14.17 (6.34 - 24.31) | 0.999 |
|  | 4 | ND - 44.35 | 14 (40.00) | 10.71 (4.43 - 16.31) | 6.05 (3.53 - 16.47) | 0.999 |
|  | | | | | | |
| IFN-α2 | Baseline | ND - 372.81 | 34 (80.95) | 31.95 (19.57 - 63.34) | 22.30 (11.82 - 41.42) | 0.24 |
|  | 2 | ND - 183.58 | 26 (63.41) | 23.02 (7.27 - 45.28) | 36.95 (10.76 - 73.99) | 0.37 |
|  | 4 | ND - 250.34 | 20 (57.14) | 42.75 (19.68 - 56.47) | 25.68 (9.06 - 58.92) | 0.82 |
|  | | | | | | |
| IFN-λ2/3 | Baseline | ND - 2112.20 | 29 (69.05) | 588.59 (388.75 - 1235.96) | 272.80 (202.65 - 512.38) | 0.05 |
|  | 2 | ND - 4330.71 | 26 (63.41) | 457.32 (53.88 - 814.74) | 62.39 (42.54 - 700.37) | 0.96 |
|  | 4 | ND - 4752.88 | 22 (62.86) | 364.13 (82.12 - 684.00) | 52.09 (35.53 - 635.92) | 0.62 |
|  | | | | | | |
| GM-CSF | Baseline | ND - 81.41 | 28 (66.67) | 16.59 (9.09 - 30.01) | 12.22 (7.98 - 16.44) | 0.26 |
|  | 2 | ND - 251.04 | 24 (58.54) | 11.97 (5.29 - 22.22) | 10.23 (7.17 - 28.13) | 0.98 |
|  | 4 | ND - 224.13 | 12 (34.29) | 48.58 (27.84 - 60.84) | 20.64 (18.27 - 32.58) | 0.27 |
|  | | | | | | |
| IFN-β | Baseline | ND - 641.96 | 36 (85.71) | 290.08 (189.21- 469.34) | 206.13 (176.99 - 321.52) | 0.29 |
|  | 2 | ND - 506.98 | 30 (73.17) | 144.97 (126.14 - 283.03) | 229.67 (32.46 - 287.04) | 0.97 |
|  | 4 | ND - 843.36 | 19 (54.29) | 259.49 (212.36 - 272.23) | 222.59 (146.11 - 342.01) | 0.72 |
|  | | | | | | |
| IL-10 | Baseline | ND - 174.16 | 41 (97.62) | 33.55 (19.98 - 82.21) | 23.43 (17.67 - 54.67) | 0.23 |
|  | 2 | ND - 158.44 | 40 (97.56) | 16.57 (10.15 - 35.97) | 15.12 (6.59 - 34.11) | 0.64 |
|  | 4 | ND - 106.55 | 34 (97.14) | 13.71 (8.55 - 19.34) | 11.06 (8.09 - 30.22) | 0.72 |
|  | | | | | | |
| IFN-γ | Baseline | ND - 1443.09 | 35 (83.33) | 259.32 (167.70 - 440.28) | 121.97 (70.32 - 211.81) | 0.03 |
|  | 2 | ND - 761.07 | 21 (51.22) | 163.60 (78.02 - 352.04) | 223.72 (98.55 - 559.30) | 0.43 |
|  | 4 | ND - 701.25 | 32 (91.43) | 143.79 (11.13 - 307.80) | 25.44 (9.19 - 228.17) | 0.79 |
|  | | | | | | |

*P-value* *corresponds to the Mann-Whitney U test comparing the average change in cytokine levels across the treatment groups.*

*ND=Non-Detectable*

**ND=Number detectable, represents cytokine concentrations detected above the lower limit of detection (LoD) of the LEGENDplex™ Multi-Analyte Flow Assay kit.*

| **Table 6. Relative hazard of RIC versus sham on study outcomes** | | |
| --- | --- | --- |
|  | | |
| **Outcome** | **Hazard ratio of RIC vs sham, (95% Cl)** | **p-value** |
| Primary outcome of death or deterioration | 1.19 (0.616 - 2.295) | *0.61* |
| Death | 1.35 (0.659 - 2.776) | *0.41* |
| Discharge | 1.66 (0.938 - 2.948) | *0.08* |
